# Unveiling the hidden role of the interaction between CD36 and FcγRIIb: implications for autoimmune disorders

**DOI:** 10.1186/s11658-024-00593-7

**Published:** 2024-05-18

**Authors:** Chenfei He, Guoying Hua, Yong Liu, Shuijie Li

**Affiliations:** 1 Center for Research in Animal Genomics, Agricultural Genome Institute at Shenzhen, Chinese Academy of Agricultural Sciences, Shenzhen, China; 2 State Key Laboratory of Frigid Zone Cardiovascular Diseases (SKLFZCD), Department of Biopharmaceutical Sciences, College of Pharmacy, Harbin Medical University, Harbin, China; 3 Heilongjiang Province Key Laboratory of Research On Molecular Targeted Anti-Tumor Drugs, Harbin, China; 4 Department of Microbiology, Tumor and Cell Biology, Karolinska Institutet, Stockholm, Sweden; 5 Department of Medical Biochemistry and Biophysics, Karolinska Institutet, Solna Campus, Stockholm, Sweden

**Keywords:** Apoptotic cell, Autoreactive B cell, FcγRIIb, Germinal center, Scavenger receptor CD36

## Abstract

**Background:**

The role of the scavenger receptor CD36 in cell metabolism and the immune response has been investigated mainly in macrophages, dendritic cells, and T cells. However, its involvement in B cells has not been comprehensively examined.

**Methods:**

To investigate the function of CD36 in B cells, we exposed *Cd36*^fl/fl^*MB1*^cre^ mice, which lack CD36 specifically in B cells, to apoptotic cells to trigger an autoimmune response. To validate the proteins that interact with CD36 in primary B cells, we conducted mass spectrometry analysis following anti-CD36 immunoprecipitation. Immunofluorescence and co-immunoprecipitation were used to confirm the protein interactions.

**Results:**

The data revealed that mice lacking CD36 in B cells exhibited a reduction in germinal center B cells and anti-DNA antibodies in vivo. Mass spectrometry analysis identified 30 potential candidates that potentially interact with CD36. Furthermore, the interaction between CD36 and the inhibitory Fc receptor FcγRIIb was first discovered by mass spectrometry and confirmed through immunofluorescence and co-immunoprecipitation techniques. Finally, deletion of FcγRIIb in mice led to decreased expression of CD36 in marginal zone B cells, germinal center B cells, and plasma cells.

**Conclusions:**

Our data indicate that CD36 in B cells is a critical regulator of autoimmunity. The interaction of CD36-FcγRIIb has the potential to serve as a therapeutic target for the treatment of autoimmune disorders.

**Supplementary Information:**

The online version contains supplementary material available at 10.1186/s11658-024-00593-7.

## Introduction

B cells play a crucial role in regulating the pathogenic mechanism of autoimmunity [1, 2], which is influenced by both central and peripheral B-cell tolerance [3]. Breaking B-cell tolerance leads to the production of autoreactive plasma cells (PC) and memory B cells and the formation of autoantibodies and autoimmune complexes [4, 5]. Systemic lupus erythematosus (SLE) is a systemic autoimmune disease that leads to the build-up of autoimmune complexes and the development of multiple organ disorders. B cells derived from SLE patients exhibit dysregulation of various cell signaling pathways. This dysregulation is characterized by the upregulation of inflammatory cytokines, specifically interleukin-6 (IL-6), and the downregulation of inhibitory receptors, including FcγRIIb, the adhesion molecule CD52, and the inhibitory cytokine IL-10 [6–9]. Therefore, there is a need to discover additional molecules relevant to autoimmunity to advance SLE therapy.

The CD36 is an 88 kDa glycoprotein that belongs to the class B scavenger receptor family [10, 11] and is found on both the cell surface and in the cytoplasm. Membrane CD36 predominantly exists as a monomer, while soluble CD36 in the cytosol is in a dimerized form [12]. CD36, a pattern recognition receptor, plays a crucial role in initiating the innate immune response. The binding ligands include a variety of substances, such as apoptotic cells, native or modified lipoproteins, glycated proteins, thrombospondin-1, and long-chain fatty acids [6]. In mice, CD36 is expressed predominantly in marginal zone B cells (MZB), followed by follicular B cells and B1 B cells [13]. CD36 expressed in MZB facilitates interactions with circulating antigens in the spleen and lymph nodes, thereby enabling the capture of antigens, including self-antigens [14]. Additionally, SLE patients exhibit serum antibodies against CD36, which leads to the accumulation of apoptotic cells and the promotion of proinflammatory responses [15]. The presence of CD36 is potentially associated with SLE development, although the exact relationship has yet to be investigated.

FcγRIIb, an inhibitory Fc receptor for IgG, modulates innate and adaptive immune responses. It is expressed predominantly in B cells and is essential for maintaining immune tolerance. FcγRIIb-deficient mice exhibit various symptoms, including collagen-induced arthritis, immune complex-mediated alveolitis, and antibody-mediated nephritis [7–9, 13]. These mice are commonly utilized as an autoimmune model. In the context of cell signaling, the co-ligation of FcγRIIb triggers the phosphorylation of the immunoreceptor tyrosine-based inhibitory motif (ITIM) domain through the action of Lyn, a member of the Src family kinase [14, 16]. Subsequent binding of SHIP1 results in the dephosphorylation of downstream molecules and inhibition of the activating signaling cascade [17, 18]. Currently, no available report has investigated the potential correlation between CD36 and FcγRIIb.

Previous studies have reported that CD36-LC3B in B cells initiates autophagy and mediates the humoral immune response [19]. Our lab published an abstract describing that CD36 co-localized with FcγRIIb after cross-linking FcγRIIb in a mouse B-cell lymphoma line, CH27, by total internal reflection fluorescence as well [20]. CD36 could uptake autoantigens, including apoptotic cells and oxidized LDL, thereby prompting inquiries into the potential involvement of CD36 in B cells in the pathogenesis of autoimmune diseases. To assess the validity of this hypothesis, we utilized an apoptotic cell-induced autoimmune model and observed that mice deficient in CD36 in B cells exhibited a compromised autoimmune response. We conducted mass spectrometry analysis and successfully identified the interaction between CD36 and FcγRIIb for the first time. Moreover, in vivo experiments showed that the loss of FcγRIIb resulted in decreased CD36 expression in B cells. Moreover, in vivo experiments have demonstrated that FcγRIIb deficiency leads to reduced CD36 expression in B cells. This study provides evidence for the potential application of the B-cell CD36-FcγRIIb interaction in autoimmunity.

## Materials and methods

### Animal models

*Cd36*KO, *Fgr2b*KO, and *Cd36*^fl/fl^ mice were obtained from Maria Febbraio (Lerner Research Institute, USA), Jeffrey V. Ravetch (Rockefeller University, USA), and Nada Abumrad (Washington University, USA), respectively. *Mb1*Cre mice (B6. C(Cg)-*Mb1*atm1 (cre) Reth/EhobJ) were acquired from the Jackson Laboratory. The control littermates of the wild type (C57BL/6) were bred from the same backcross. The mice used in each experiment were matched for age (8–12 weeks) and gender.

Thymocytes derived from 4–5-week-old C57BL/6 mice were cultured in a medium containing 1 μM dexamethasone (Sigma-Aldrich) for 12 h to obtain apoptotic cells. To establish an induced autoimmune mouse model, 20 × 10^6^ apoptotic cells were intravenously injected [21]. Mice were administered injections weekly four times. Animals were maintained and bred in a controlled environment free of pathogens at the Center for Molecular Medicine L8 animal facility. All the experimental procedures were approved by the local ethical committee, specifically the North Stockholm district court.

### Enzyme-linked immunosorbent assay

Enzyme-linked immunosorbent assay (ELISA) plates were coated with 5 µg/mL methylated BSA (Sigma-Aldrich) in phosphate-buffered saline (PBS), followed by 50 µg/mL calf thymus DNA (Sigma-Aldrich) in PBS. The coated plates were treated with a blocking solution containing 10% fetal bovine serum (FBS) (HyClone) in PBS at room temperature (RT) for 2 h. The plates were incubated with diluted mouse serum at RT for 1 h. Following this, the plates were coated with horseradish peroxidase (HRP)-conjugated secondary antibodies (SouthernBiotec) at RT for 1 h. Finally, the plates were developed using a TMB substrate (BioLegend). The optical density at 450 nm (OD 450) and 620 nm (OD 620) was measured using an Eon Microplate Spectrophotometer (BioTek).

### The binding activity of B cells to apoptotic cells

Freshly isolated mouse thymocytes were labeled with the red fluorescent cell membrane dye PKH26 (Sigma-Aldrich) following the manufacturer's instructions. Subsequently, cell apoptosis was induced by 1 μM dexamethasone (Sigma-Aldrich) overnight and labeled with PKH26. To examine the binding ability of B cells to apoptotic cells, we co-cultured B cells with PKH26-labeled apoptotic cells at 37 °C for 2 h. Cells were collected for fluorescent detection using flow cytometry.

### Flow cytometry

To minimize nonspecific binding, cells were treated with purified anti-mouse CD16/32 (BD) for Fc-blocking. Additionally, a live/dead fixable stain kit (Invitrogen) was used to exclude dead cells during staining. The samples were subjected to staining with surface antibodies for 30 min at 4 °C. The surface antibodies used for flow cytometry analysis included PE CD1d (BioLegend), PE-Cy7 CD95 (BD), PE-Cy7 CD138 (BioLegend), BV421 B220 (BD), V500 B220 (BD), APC IgD (BioLegend, 405714), FITC anti-GL-7 (BD), and AF700 CD36 (Bio-Rad). The samples were detected using flow cytometry (BD, LSRFortessaTMX-20) and subsequently analyzed using FlowJo software.

### Purification and activation of primary B cells

To isolate primary mouse B cells, the spleens of the mice were harvested, filtered, and subjected to red blood cell lysis. The negatively selected B cells were isolated using the EasySep™ Mouse B-Cell Isolation Kit (Stemcell™ Technologies). Purified B cells were treated with 10 μg/mL LPS (Sigma-Aldrich) or 1 μM CpG (InvivoGen) for three days. Primary B and CH12 cells were cultured at a density of 1 × 10^6^ cells/mL in RPMI medium (Gibco™) supplemented with 10% FBS (HyClone), 1 × penicillin–streptomycin (HyClone), 1 mM sodium pyruvate (Sigma-Aldrich) and 50 μM 2-mercaptoethanol (Gibco™). The cells were cultured in a humidified environment at 37 °C with 5% CO2. To generate *Cd36* knockout CH12 cells (CH12*Cd36*KO), the plasmids pPB-CRISPR-gRNA and CAG-hyPbase were co-transfected into CH12 cells using a mouse B-cell nucleofector™ Kit (Lonza) [19, 22]. One week following the transfection, individual cell-derived clones were selected and subjected to analysis via Sanger sequencing.

### Confocal microscopy

For immunofluorescence microscopy, B cells were cultured on glass coverslips coated with poly-L-lysine and fixed with cold methanol for 20 min. The samples were treated with AF488-conjugated anti-CD36 antibodies (Bio-Rad Laboratories) and PE-conjugated anti-CD16/CD32 antibodies (BD) in a solution containing 1% FBS in PBS. The mixture was incubated overnight at 4 °C. The washed slides were mounted using ProLong™ Diamond Antifade Mountant (Invitrogen). Visual data were obtained using a confocal microscope (Zeiss, LSM880) and subsequently analyzed using LSM image software.

### Immunoblotting and coimmunoprecipitation

For the immunoblotting and immunoprecipitation assays, the cells were lysed using a buffer solution consisting of 0.5% NP-40 (Sigma), 10 mM Tris–HCl (Sigma), 150 mM NaCl (Sigma), protease inhibitor cocktail (Sigma), and phosphatase inhibitor cocktail 2 (Sigma). The soluble protein concentration was quantified using the DC™ Protein Assay Kit (Bio-Rad). To conduct immunoprecipitation, a total of 2 µg of goat anti-mouse CD36 antibody (R&D) or normal goat IgG (Millipore) was incubated with Pierce™ Protein A/G Magnetic Beads (Thermo Scientific™) overnight. Dynabeads were crosslinked with antibodies using 250 µL of a 5 mM solution of BS3 (bis(sulfosuccinimidyl) suberate) (Thermo Scientific™) and subsequently coated with the soluble proteins overnight. Immunoprecipitated proteins were resolved using 4–12% Bis–Tris gradient gels (Life Technologies) and transferred onto a nitrocellulose membrane using the Trans-Blot® Turbo™ Transfer System (Bio-Rad). The blots were transferred and blocked with a 5% milk solution in PBST. This was followed by incubation with both primary and secondary antibodies. The blots were developed using enhanced ECLTM Prime (Amersham Pharmacia Biotech) and subsequently scanned using an ImageQuant LAS4000 system (Amersham). The band densitometry analysis was performed using ImageJ software. The primary and secondary antibodies used for immunoprecipitation and immunoblotting were as follows: Mouse CD36/SR-B3 Antibody (R&D), FcγRIIb rabbit mAb (CST), Rabbit IgG isotype (CST), goat IgG isotype (R&D), conjugated sheep anti-rabbit IgG-HRP (Millipore), and mouse anti-goat IgG-HRP (Santa Cruz Biotechnology).

### Liquid chromatography coupled to tandem mass spectrometry (LC–MS/MS)

To facilitate mass spectrometry analysis, samples were prepared for mass spectrometry analysis by a modified version of the filter-aided sample preparation protocol digestion [23]. The protein samples, each containing 200 µg of protein, were combined with 1 mM DTT, 8 M urea, and 25 mM HEPES (pH 7.6). This mixture was centrifuged using a filtering unit with a 10 kDa cutoff, specifically a Nanosep® Centrifugal Devices with Omega™ Membrane 10 K (Pall). The samples were centrifuged at a force of 14,000 × g for 15 min, after which the resulting flow-through was discarded. Following this, a subsequent iteration of the 8 M urea buffer was employed, followed by centrifugation. Proteins were subjected to alkylation by adding 25 mM IAA in a solution containing 8 M urea and 25 mM HEPES at pH 7.6 for 10 min. The resulting mixture was then centrifuged at 14,000 × g for 15 min. This process was repeated twice, with a buffer of 8 M urea and 25 mM HEPES at pH 7.6, followed by centrifugation. The flow-through was discarded after each centrifugation step.

Protein samples were subjected to digestion on the filter employing Pierce™ Trypsin Protease (Thermo Scientific) at a final ratio of 1:50 (protease to protein) in 50 mM HEPES buffer at pH 7.6. The samples were then incubated overnight at 37 °C. After digestion, the filter units were centrifuged at a speed of 14,000 × *g* for 15 min. The resulting flow-through was collected and subsequently subjected to another round of centrifugation, 50 µL of Milli-Q water. The flow-through from this second centrifugation step was also retained. Before labeling, the pH was adjusted using 1 M triethylammonium bicarbonate buffer to achieve a pH of 8.5 (with a final concentration of 100 mM). Eighty micrograms of peptide were labeled using the TMT10plex™ Isobaric Label Reagent Set (Thermo Scientific) following the manufacturer's protocol. The peptides were subjected to an additional desalting process using Strata-X solid-phase extraction (Phenomenex) for reverse-phase extraction. The purified samples were dried using a SpeedVac and subsequently stored at -20 °C. The samples were dissolved in 20 µL of solvent A, and 10 μL was injected.

Samples were captured using a C18 guard-desalting column (Acclaim PepMap 100, 75 μm × 2 cm, nanofiber, C18, 5 µm, 100 Å) and subsequently separated on a 50 cm long C18 column (EasySpray PepMap RSLC, C18, 2 μm, 100 Å, 75 μm × 50 cm). The composition of solvent A used in the nanocapillary was 99.9% water and 0.1% formic acid, while solvent B consisted of 5% water, 95% acetonitrile, and 0.1% formic acid. The sample was detected using a mass spectrometer. The MS raw files were analyzed using the Sequest HT-Target Decoy PSM Validator within the Proteome Discoverer 1.4 software platform (Thermo Scientific). The search was performed against the human Swiss-Prot database (released in March 2019), and the results were filtered using a 1% false discovery rate (FDR) cutoff.

### Statistical analysis

Prism software version 8.0 was utilized for the analysis of the graphs and data. Statistical significance was assessed using the Mann‒Whitney test, with significance levels denoted as *P < 0.05, **P < 0.01, and ***P < 0.001.

## Results

### The scavenger receptor CD36 plays a crucial role in the B-cell-mediated immune response against autoantigens

To assess the role of CD36 in vitro, we measured the binding ability of B cells to apoptotic cells labeled with fluorescent PKH26. There were significantly fewer CPG-initiated B cells from the *Cd36*KO mice than from the WT mice (Fig. S1A). Moreover, CH12 B lymphoma cells lacking CD36 had fewer cells attached to apoptotic cells (Fig. S1B), which is consistent with the pattern observed in primary B cells. Taken together, these results indicate that CD36 is essential for transporting apoptotic B cells.

**Fig. 1:**
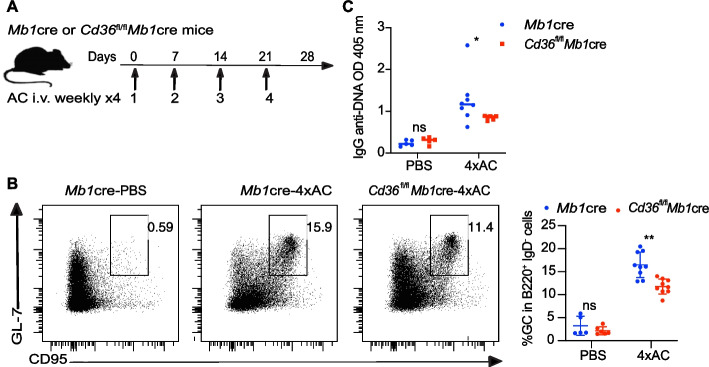
Mice that lack CD36-expressing B cells exhibit compromised autoimmunity. **A** The immunization strategy employed for the *Mb1*cre and *Cd36*^fl/fl^*Mb1*cre mice. Mice were immunized four times weekly with apoptotic cells (4xAC), while PBS was used as the negative control. **B** The gating strategy and frequency of germinal center (GC) B cells were examined. **C** Measurement of anti-DNA IgG levels via ELISA. The data are representative of three independent experiments. *P < 0.05, **P < 0.01 and ***P < 0.001 (Mann–Whitney)

The accumulation of self-DNA can break self-tolerance. We previously reported that multiple intravenous injections of apoptotic cells result in SLE-like disorders in mice [24]. The production of autoimmune antibodies is used as the primary indicator in this induced autoimmune model. In this study, apoptotic cells were administered weekly four times to induce an autoimmune response in mice. To investigate the role of CD36 in vivo, we conducted a crossbreeding experiment between mice harboring floxed CD36 alleles (*Cd36*^fl/fl^) and mice harboring MB1cre **(**Fig. 1A). This resulted in the generation of *Cd36*^fl/fl^*Mb1*cre mice, which have a specific knockout of CD36 in B cells [25, 26]; the *Mb1*cre mice served as the controls. To assess the efficacy of CD36-specific knockout in B cells, we analyzed CD36 expression in marginal zone B cells (MZB), which exhibit the highest level of CD36 among all B cell subsets[19], by comparing *Cd36*^fl/fl^*Mb1*cre and *Mb1*cre mice. The proportion of CD36-positive cells in MZB reaches 86.9% in *Mb1*cre mice, while only 5.0% CD36^+^ MZB showed in *Cd36*^fl/fl^*Mb1*cre mice. This means that 94.2% of CD36^+^ MZB cells have been deleted in *Cd36*^fl/fl^*Mb1*cre mice (Fig. S1C). The data suggests that *Cd36*^fl/fl^*Mb1*cre mice exhibit CD36-specific knockout in B cells.

Here, to evaluate the autoimmune response in mice lacking CD36 B cells, we detected the formation of GC B cells in the spleen and the production of autoantibodies against DNA in the blood serum. Without immunization, the formation of germinal center B cells was only approximately 1–2%. In contrast, during apoptotic cell immunization, the frequency of germinal center B cells reached 11% in the *Cd36*^fl/fl^*Mb1*cre mice but 15% in the control group (Fig. 1B and Fig. S1D). The level of IgG against DNA was very low, and no difference was found between *Cd36*^fl/fl^*Mb1*cre mice and controls at steady state. In the apoptotic cell-induced model, we observed a reduction in the serum levels of anti-DNA IgG antibodies in *Cd36*^fl/fl^*Mb1*cre mice compared to those in the control group (Fig. 1C). These data suggest that CD36 in B cells is necessary for a sufficient germinal center response and to produce autoantibodies in an autoimmune-induced model.

### Protein candidates interact with CD36 in primary B cells

**Fig. 2:**
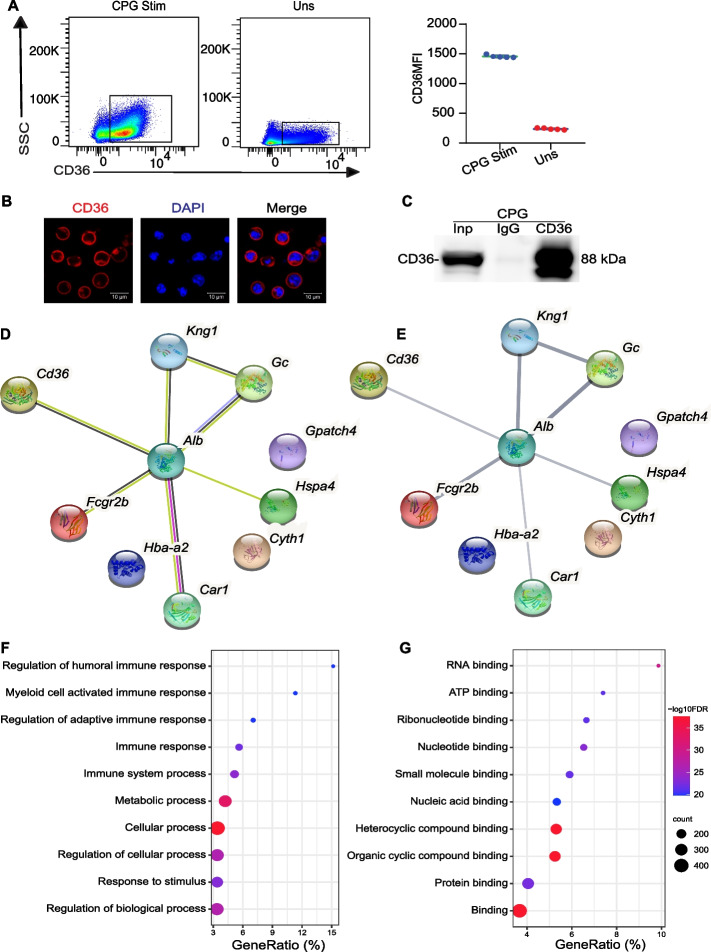
CD36-associated proteins were identified through co-immunoprecipitation followed by mass spectrometry analysis. **A** The pseudocolor plot illustrates the staining of CD36 in B cells that were either stimulated with CPG (CPG Stim) or unstimulated (Uns). **B** Confocal microscopy was used to analyze CD36 staining in B cells treated with CPG. **C** Immunoblot analysis of soluble proteins derived from B cells stimulated with CPG was subsequently performed using an anti-CD36 antibody. The total B cell protein served as the loading (positive) control referred to as Input (Inp). The B cell protein was immunoprecipitated using isotype (negative) control IgG antibodies (IgG) or anti-CD36 antibodies (CD36). **D** Evidence of the CD36 protein network predicted by the STRING, demonstrating its functional association with the top ten proteins. Network nodes represent proteins. Colored nodes represent the query protein and its first shell of interactors, while white nodes represent the query protein and the second shell of interactors. Filled nodes or empty nodes indicate predicted or unpredicted 3D structures, and edges represent protein–protein associations. Line color indicates the different types of interaction evidence. **E** Confidence view of the CD36 protein network. The line of thickness visually depicts stronger associations. **F**, **G** Gene Ontology enrichment analysis was also conducted to investigate the biological processes and molecular functions associated with the proteins identified through anti-CD36 immunoprecipitation. The data are representative of three independent experiments. *P < 0.05, **P < 0.01 and ***P < 0.001 (Mann–Whitney)

Synthetic unmethylated cytidine phosphate guanosine (CpG) oligodeoxynucleotides, referred to as CpG, are a widely used agonist for TLR9. It stimulates various antigen-presenting cells, initiating innate and adaptive immune responses [27]. To observe the distribution of CD36 in B cells, we stimulated primary B cells in mice with the 1 μM TLR9 agonist CPG. We found that CD36 expression in B cells was upregulated more than fivefold in response to CPG stimulation compared to that of controls (Fig. 2A). Moreover, immunofluorescence staining with an anti-CD36 antibody revealed that CD36 expression in cells treated with CPG was present on the B-cell membrane and in the cytosol (Fig. 2B). This finding is consistent with our previous discovery [19]. To screen out the interacted protein candidates with CD36 in primary B cells activated by CPG, we conducted mass spectrometry analysis after immunoprecipitation with an anti-CD36 antibody following the workflow outlined in Fig. S2. CD36 enrichment was observed after B-cell immunoprecipitation, as depicted in Fig. 2C. It suggests that anti-CD36 antibodies successfully immunoprecipitated CD36 and its interacting proteins in CPG stimulated B cells.

We identified 30 potential protein candidates. Protein–protein interaction networks were predicted using the STRING version 11.5 program, which was accessed at https://cn.string-db.org/. In this study, we presented the potential associations between CD36 and the top 10 proteins, including FcγRIIb, CYTH1, HSPA4, GPATCH4, KNG1, ALB, GC, CA1, HBA, and LDHA (Fig. 2D, E). To assess the correlation between the candidates, we analyzed both the biological process and the molecular function of the genes. Most protein candidates play crucial roles in regulating biological processes, responses to stimuli, cellular processes, metabolic processes, and immune responses (Fig. 2F). In terms of molecular function, most identified proteins exhibited the ability to bind with other proteins, organic cyclic compounds, nucleic acids, and other molecules. (Fig. 2G). In summary, the data indicate that protein candidates that interact with CD36 play a role in regulating biological processes and demonstrate binding ability in B cells.

### Interaction of CD36 with FcγRIIb in primary B cells activated by CPG

**Fig. 3:**
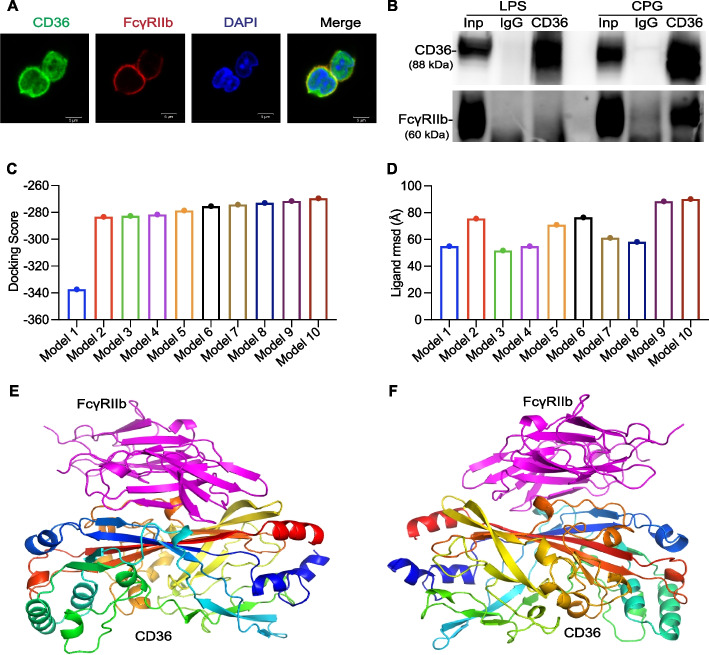
The protein interaction between CD36 and FcγRIIb was confirmed through various experimental approaches. **A** CD36 and FcγRIIb staining analysis of CPG-stimulated B cells using confocal microscopy. **B** The proteins immunoprecipitated by anti-CD36 antibodies were detected using anti-FcγRIIb antibodies in B cells stimulated with cytidine phosphate guanosine (CPG) or lipopolysaccharide (LPS). The total B cell protein served as the loading (positive) control referred to as Input (Inp). The B cell protein was immunoprecipitated using isotype (negative) control IgG antibodies (IgG) or anti-CD36 antibodies (CD36). **C**,** D** Docking scores and ligand mean square deviation (rmsd) for the models of CD36 docking with FcγRIIb by HDOCK SERVER. The X-axis represents the rank of the top 10 models, and the Y-axis shows the docking energy scores in **C**. The ligand rmsd is calculated by comparing the ligands in the CD36 model with the modeled FcγRIIb structures in **D**. **E**, **F** The front and back sides of the model 1 of CD36 (multicolor) docking with FcγRIIb (purple)

According to the mass spectrometry analysis, FcγRIIb was identified as the top candidate for CD36 interaction and was chosen to validate the reliability of the method. To validate the interaction between CD36 and FcγRIIb, we conducted both immunofluorescence and immunoprecipitation analyses to evaluate the colocalization and co-immunoprecipitation potential of these two proteins in CPG-activated primary B cells. After staining with anti-CD36 and FcγRIIb antibodies, fluorescence confocal microscopy revealed that a portion of the CD36 membrane was colocalized with FcγRIIb on the surface of the B cells (Fig. 3A). Moreover, protein enrichment by anti-CD36 antibodies revealed the interaction of FcγRIIb with CD36 under CPG (TLR9 agonist) stimulation but not under lipopolysaccharide (LPS), TLR4 agonists, stimulation (Fig. 3B). For immunoprecipitation mediated by anti-FcγRIIb antibodies, the data showed that anti-FcγRIIb antibodies specifically immunoprecipitated protein FcγRIIb compared to the isotype control. Unfortunately, we did not detect protein CD36 in the co-immunoprecipitated proteins (Fig. S3A). CD36-FcγRIIb interaction was confirmed through immunofluorescence co-localization and co-immunoprecipitation.

The docking of CD36-FcγRIIb was analyzed using the HDOCK SERVER [28] which utilizes a hybrid algorithm for template-based modeling (http://hdock.phys.hust.edu.cn/). The docking characteristics were evaluated, including complex template information, docking score, and ligand root mean square deviation (rmsd). We used 5LGD and 2FCB from Protein Data Bank (PDB) (https://www.rcsb.org/) as templates for CD36 and FcγRIIb, respectively (Table 1). The prediction of protein–protein binding showed the top 10 models of CD36 docking with FcγRIIb (Fig. S3B). These binding models of CD36-FcγRIIb interaction exhibited various docking energy scores and ligand rmsd (Fig. 3C, D). Model 1 revealed the best docking performance, with the lowest docking score and a relatively low rmsd value. Both the anterior and superior aspects of Model 1 revealed numerous interaction sites between CD36 and FcγRIIb (Fig. 3E, F)**.** We analyzed the interface residue pairs and rmsd in Model 1 of CD36 docking with FcγRIIb and found 100 interface residue pairs within 5.0 Å (Table 2). Model 1 of CD36-FcγRIIb interaction outperformed the other models predicted by HDOCK SERVER.

**Table 1:** Complex template information for CD36 and FcγRIIb

Molecule	PDB ID	Chain ID	Align_length	Coverage	Seq_ID (%)
CD36	5LGD	A	472	1	84.1
FcγRIIb	2FCB	A	170	0.563	64.7

The Protein Data Bank (PDB) IDs are from PDB. This table contains the chain ID, aligned sequence length, coverage of aligned sequence, and sequence identity between CD36 and FcγRIIb

**Table 2:** Interface residue pairs and ligand root mean square deviation (rmsd) (Å) for Model 1 of CD36 docking with FcγRIIb

Interface residue pairs	Ligand rmsd (Å)	Interface residue pairs	Ligand rmsd (Å)	Interface residue pairs	Ligand rmsd (Å)
60A-162A	3.877	92A-161A	2.84	363A-137A	3.445
62A-161A	4.158	125A-160A	4.251	364A-42A	4.192
62A-162A	3.852	126A-159A	4.53	365A-51A	4.275
64A-159A	4.65	126A-160A	3.059	365A-137A	3.366
64A-161A	2.512	126A-161A	3.593	365A-139A	3.583
64A-164A	1.685	126A-162A	4.122	365A-143A	3.163
64A-165A	4.713	127A-159A	4.609	366A-44A	3.366
64A-166A	4.742	128A-159A	2.211	366A-137A	3.266
66A-164A	4.038	128A-168A	3.691	366A-166A	3.712
66A-166A	3.567	128A-170A	4.844	368A-143A	4.047
66A-168A	4.248	281A-142A	3.952	370A-145A	2.644
69A-171A	4.089	282A-142A	2.397	370A-163A	1.654
71A-131A	4.93	283A-142A	4.968	372A-145A	2.75
71A-171A	3.602	283A-143A	4.089	382A-145A	1.293
86A-131A	3.593	283A-144A	3.11	382A-146A	4.215
86A-132A	3.142	283A-145A	3.167	382A-163A	2.157
86A-133A	3.051	284A-186A	4.859	383A-163A	3.853
86A-171A	3.867	285A-145A	2.836	384A-163A	4.333
87A-133A	2.75	286A-147A	3.166	415A-166A	3.978
87A-135A	2.72	286A-185A	4.306	417A-163A	4.978
87A-168A	4.305	286A-187A	3.716	417A-164A	4.95
88A-133A	4.606	286A-188A	3.355	419A-163A	2.671
88A-168A	3.827	287A-188A	3.344	419A-164A	4.799
88A-170A	3.381	292A-143A	2.942	420A-163A	4.292
89A-159A	3.388	292A-144A	3.936	421A-147A	4.698
89A-167A	4.541	292A-145A	2.751	421A-162A	3.981
89A-168A	1.668	361A -41A	4.851	421A-163A	2.733
89A-169A	4.868	361A-43A	3.072	424A-147A	4.62
89A-170A	4.376	362A-41A	3.713	424A-148A	4.264
90A-159A	2.416	362A-42A	3.711	424A-187A	4.865
90A-164A	4.338	362A-43A	3.653	428A-188A	3.47
90A-166A	3.477	363A-42A	1.952	431A-188A	3.785
90A-167A	4.243	363A-43A	4.742		
90A-168A	3.706	363A-44A	2.994		

The interface information includes all residue pairs within ligand rmsd of 5.0 Å

### Deletion of FcγRIIb limits CD36 expression in vivo

**Fig. 4:**
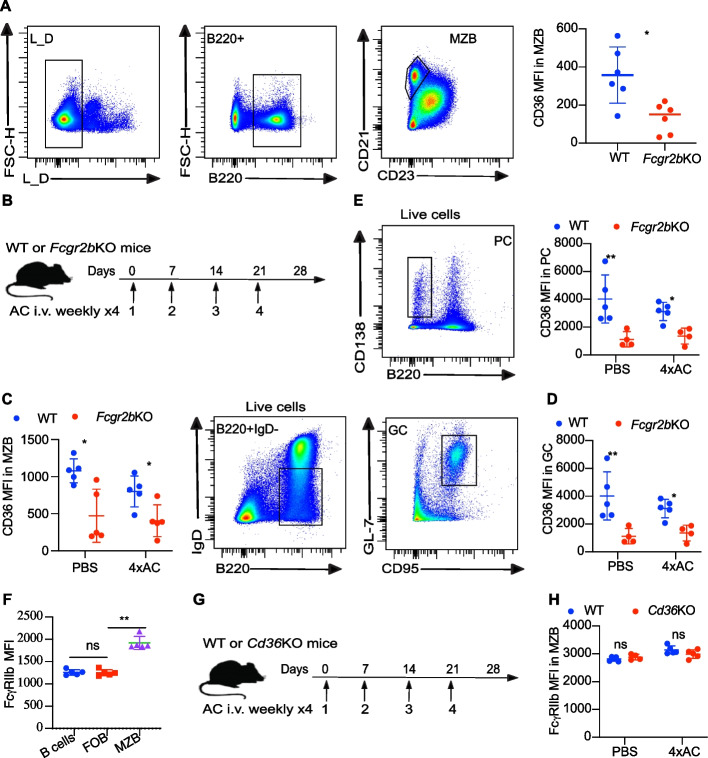
Loss of FcγRIIb in mice downregulates CD36 expression in B cells. **A** The gating strategy and scatter plot depict CD36 staining in marginal zone B cells (MZB) (CD19^+^CD21^+^CD23^mid^) from both wild-type (WT) and *Fcgr2b* knockout (*Fcgr2b*KO) mice determined via flow cytometry. **B** The immunization strategy involved apoptotic cells (4xAC) in WT and *Fcgr2b*KO mice. PBS served as the negative control. **C** The CD36 staining was performed on MZB in WT and *Fcgr2b*KO mice under injections. **D**, **E** The gating strategy and scatter plot illustrate the CD36 staining pattern in germinal center B cells (GC) (B220^+^IgD^−^CD95^+^GL-7^+^) of and plasma cells (PC) (B220^int^CD138^+^) in immunized WT and *Fcgr2b*KO mice. **F** The FcγRIIb staining on B cells, follicular B cells (FOB), and marginal zone B cells (MZB). **G** The immunization strategy was used for both WT and *Cd36* knockout (*Cd36*KO) mice with four times weekly apoptotic cells (4xAC) or PBS. **H** The FcγRIIb staining of MZB in WT and *Cd36*KO mice after treatments. The data are representative of three independent experiments. *P < 0.05, **P < 0.01 and ***P < 0.001 (Mann–Whitney)

To examine the impact of FcγRIIb on the expression of CD36, we used *Fcgr2b* knockout (*Fcgr2b*KO) mice to assess the CD36 level in the B-cell subsets. In the context of the steady state, the absence of FcγRIIb led to reduced CD36 expression in marginal zone B cells in mice (Fig. 4A). Moreover, in both the injection of PBS and apoptotic cells, mice lacking FcγRIIb exhibited lower CD36 levels in marginal zone B cells, germinal center B cells, and plasma cells compared with control mice (Fig. 4B-E). These data indicate that the presence of FcγRIIb influences the surface expression of CD36 in B cell subsets, such as marginal zone B cells, germinal center B cells, and plasma cells, in both steady and autoimmune induction scenarios.

To characterize the expression of FcγRIIb in B cell subsets, we investigated its expression in various B cell subsets, including total B cells, follicular B cells, and marginal zone B cells. FcγRIIb expression in marginal zone B cells was higher than in other B cell subsets (Fig. 4F). To further investigate the potential regulatory effect of CD36 on the FcγRIIb, we immunized *Cd36* knockout (*Cd36*KO) mice by comparing the surface FcγRIIb expression on the surface of MZB cells (Fig. 4G). We found that the expression of FcγRIIb showed no significant difference between *Cd36*KO and control mice in both steady and autoimmune-induced conditions (Fig. 4H). The absence of CD36 in mice did not influence the expression of FcγRIIb in vivo.

## Discussion

Here, we demonstrated the critical role of CD36 in the autoimmune response. Utilizing conditional gene-deficient mice, our study revealed that the expression of CD36 on B cells facilitates the uptake of apoptotic cells in the bloodstream, leading to the development of an autoimmune response. The interaction between CD36 and FcγRIIb was identified using proteomics techniques and subsequently validated through confocal microscopy and co-immunoprecipitation assays. Additionally, FcγRIIb deficiency results in decreased CD36 expression in marginal zone B cells, germinal center B cells, and plasma cells. The study concluded that the interaction between CD36 and FcγRIIb in B cells regulates the autoreactive response.

CD36 plays a crucial role in the transport of long-chain fatty acids (LCFAs), proteins that contain thrombospondin structural homology repeat (TSR) domains, and molecules that exhibit molecular structures consistent with danger-associated or pathogen-associated molecular patterns (DAMPs and PAMPs). The absence of CD36 regulates the expression of LCFAs, the TSR, and the DAMP/PAMP signaling pathways, thereby modulating both innate and adaptive immune responses [6, 29]. Our previous study demonstrated the role of CD36-mediated autophagy in the T-cell-dependent humoral immune response through the interaction of CD36-LC3b [19]. Here, we present compelling evidence supporting the critical role of CD36 in autoimmunity, specifically in the development of germinal center B cells and the production of autoantibodies.

During the co-immunoprecipitation with anti-CD36 antibodies, FcγRIIB was pulled down in CPG-stimulated B cells, while CD36 was not detected in the enriched proteins using anti-FcγRIIB antibodies. The former phenotype was observed using mass spectrometry, confocal microscopy, and co-immunoprecipitation; therefore, it provides strong evidence-based data. Unfortunately, we failed to detect protein CD36 in the enriched proteins. CD36 may act dominantly through FcγRIIB signaling, but FcγRIIB mainly functions through the inhibitory pathway, triggering the phosphorylation of SHIP-1 and SHP-1 [30]. On the other hand, some antibodies perform excellently in western blotting but poorly in pulling down the interacted candidates. Here, although anti-FcγRIIB antibodies work well in western blotting and FcγRIIB immunoprecipitation, they perform poorly in capturing the interacting proteins. Thus, it was difficult to detect the binding when we performed co-immunoprecipitation using anti-FcγRIIB antibodies.

In addition to its established inhibitory function in autoimmunity, our research revealed a potential novel role of FcγRIIB in the interaction with CD36. Interestingly, FcγRIIB on the surface of B cells was necessary for adequate expression of CD36 in vivo. FcγRIIB can regulate the activation of myeloid cells by inhibiting Toll-like receptor (TLR) 2, 3, 4, 7, and 9-dependent pathways, in addition to its role in activating FcγR [31–33]. This study revealed that FcγRIIB functions by modulating the function of CD36 through interactions between these two proteins. We hypothesize that a combination of these potential pathways may necessitate further investigations.

With and without apoptotic cell administration, mice lacking FcγRIIB exhibited less CD36 expression in the germinal center and plasma cells than wild-type mice. The presence of FcγRIIB is implicated in modulating CD36 expression in both steady and autoimmune induction states. Moreover, immunizing apoptotic cells in wild-type mice resulted in lower CD36 levels in GCs and plasma cells than in unimmunized mice. These findings suggest that the expression of CD36 in germinal centers and plasma cells is influenced by treatment with apoptotic cells. CD36 is potentially correlated with the function of CD36 in transporting apoptotic cells or B-cell metabolism [6, 19, 20].

This is a limitation of this study. For mass spectrometry, primary B cells were treated with the TLR9 agonist CPG. It would be better if we could perform proteomic analysis by using primary B cells without treatment or MZB cells isolated from apoptotic cell-immunized mice. However, objective realities restrict the conduct of experiments. On the one hand, primary B cells express a very limited level of CD36 [19, 34], so the amount of CD36 in primary B cells is not sufficient for the protein quantity required for mass spectrometry. On the other hand, the frequency of MZB cells in mice is low; therefore, it is difficult to reach the amount of B cells for mass spectrometry.

## Conclusions

Our data indicate that CD36 in B cells is a critical regulator of autoimmunity. The interaction of CD36-FcγRIIb has the potential to serve as a therapeutic target for the treatment of autoimmune disorders.

### Supplementary Information

Supplementary Material 1: Fig. S1. B cells lacking CD36 exhibit reduced binding ability to apoptotic cells. (**A**) The binding ability of PKH26-stained apoptotic cells (PKH26^+^) to CPG-stimulated B cells from WT and *Cd36* knockout (*Cd36*KO) mice. (**B**) PKH26-stained apoptotic cells (PKH26^+^) were able to bind to the CH12 and *Cd36* knockout cells (CH12KO). (**C**) Gating strategy for marginal zone B cells (MZB) (CD19^+^CD21^+^CD23^mid^) and percentage of CD36^+^ MZB in Cd36fl/flMb1cre and Mb1cre mice. (**D**) The gating strategy for identifying germinal center B cells. The data are representative of three independent experiments. *P < 0.05, **P < 0.01 and ***P < 0.001 (Mann–Whitney).

Supplementary Material 2: Fig. S2. Mass spectrometry experimental setup. CD36-interacting candidates in CPG-stimulated primary B cells were immunoprecipitated using anti-CD36 antibodies. The proteins were digested into peptides and analyzed by mass spectrometry.

Supplementary Material 3: Fig. S3. Immunoprecipitation by anti-FcγRIIb antibodies and models for the docking of CD36 with FcγRIIb. (**A**) Immunoprecipitation using anti-FcγRIIb antibodies. CPG-stimulated B cell protein served as the loading control, referred to as Input (Inp). The B cell protein was immunoprecipitated using isotype control IgG antibodies (IgG), and anti-FcγRIIb antibodies (FcγRIIb). (**B**) Models 2–10 depict the docking of CD36 with FcγRIIb on the front side. Purple and multicolor represent FcγRIIb and CD36 structure models, respectively.

## Data Availability

The data from this study are available in this published article.

## Abbreviations

AC: Apoptotic cells
CpG: Cytidine phosphate guanosine
DAMP: Danger-associated molecular pattern
ELISA: Enzyme-linked immunosorbent assay
FDR: False discovery rate
GC: Germinal center B cells
IL: Interleukin
ITIM: Immunoreceptor tyrosine-based inhibitory motif
LCFAs: Long-chain fatty acids
LC‒MS: Liquid chromatography coupled to tandem mass spectrometry
LPS: Lipopolysaccharide
MZB: Marginal zone B cells
PAMP: Pathogen-associated molecular pattern
PC: Plasma cells
rmsd: Root mean square deviation
SLE: Systemic lupus erythematosus
TLR: Toll-like receptor
TSR: Thrombospondin structural homology repeat
