# Supplementary figures and images for: Unveiling the hidden role of the interaction between CD36 and FcγRIIb: implications for autoimmune disorders
Source: Cell Mol Biol Lett. 2024 May 18;29:76. doi: 10.1186/s11658-024-00593-7 (PMC11102138; doi:10.1186/s11658-024-00593-7)

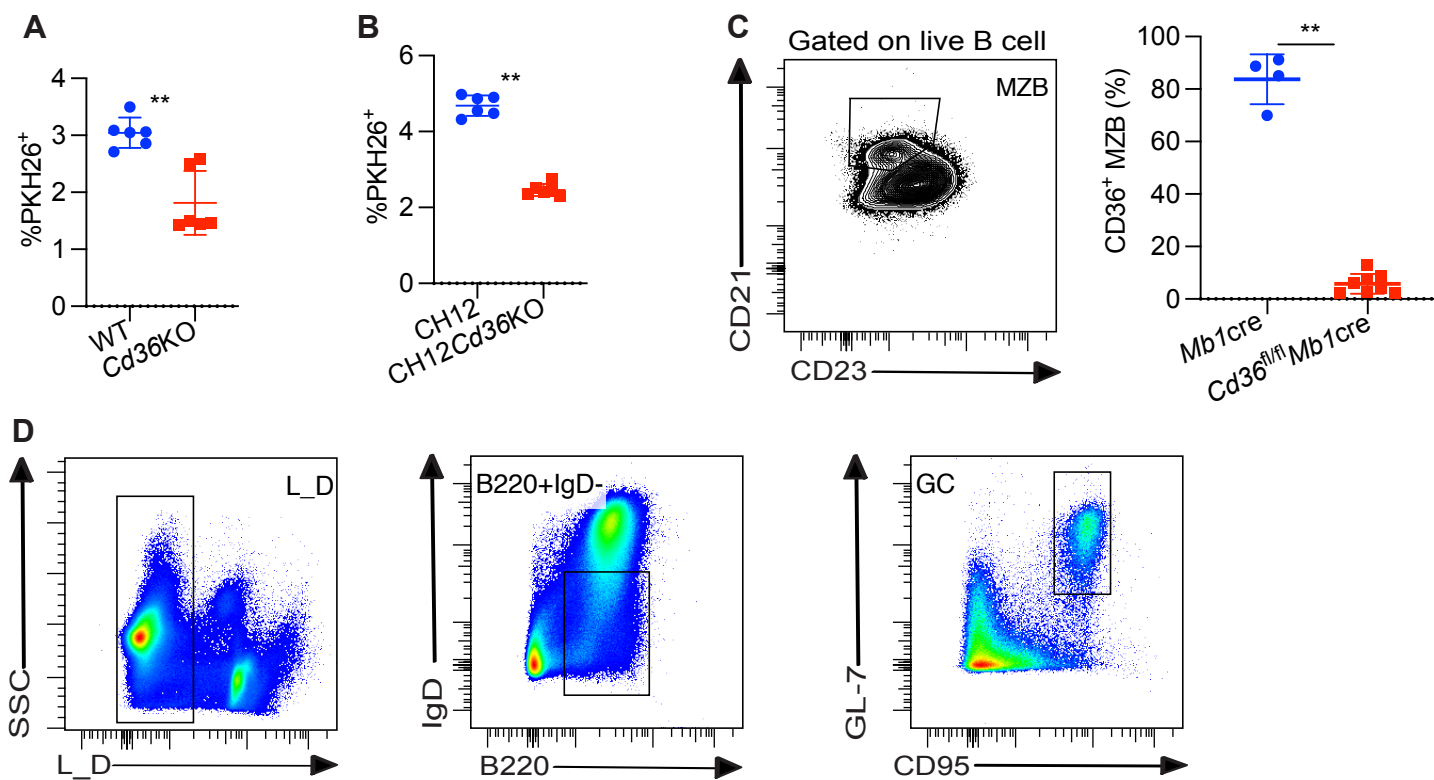

Supplement: Supplementary file 1 — Supplementary Material 1: Fig. S1. B cells lacking CD36 exhibit reduced binding ability to apoptotic cells. (A) The binding ability of PKH26-stained apoptotic cells (PKH26+) to CPG-stimulated B cells from WT and Cd36 knockout (Cd36KO) mice. (B) PKH26-stained apoptotic cells (PKH26+) were able to bind to the CH12 and Cd36 knockout cells (CH12KO). (C) Gating strategy for marginal zone B cells (MZB) (CD19+CD21+CD23mid) and percentage of CD36+ MZB in Cd36fl/flMb1cre and Mb1cre mice. (D) The gating strategy for identifying germinal center B cells. The data are representative of three independent experiments. *P < 0.05, **P < 0.01 and ***P < 0.001 (Mann–Whitney). [file 11658_2024_593_MOESM1_ESM.pdf]

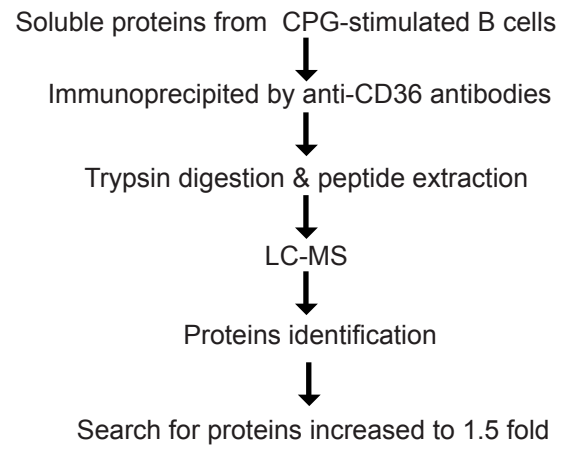

Supplement: Supplementary file 2 — Supplementary Material 2: Fig. S2. Mass spectrometry experimental setup. CD36-interacting candidates in CPG-stimulated primary B cells were immunoprecipitated using anti-CD36 antibodies. The proteins were digested into peptides and analyzed by mass spectrometry. [file 11658_2024_593_MOESM2_ESM.pdf]

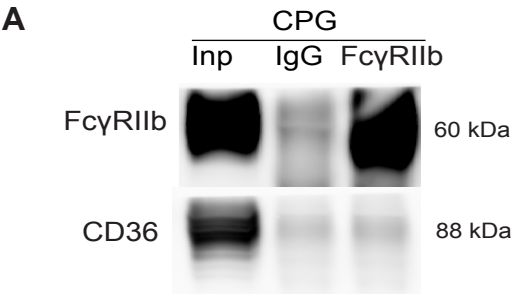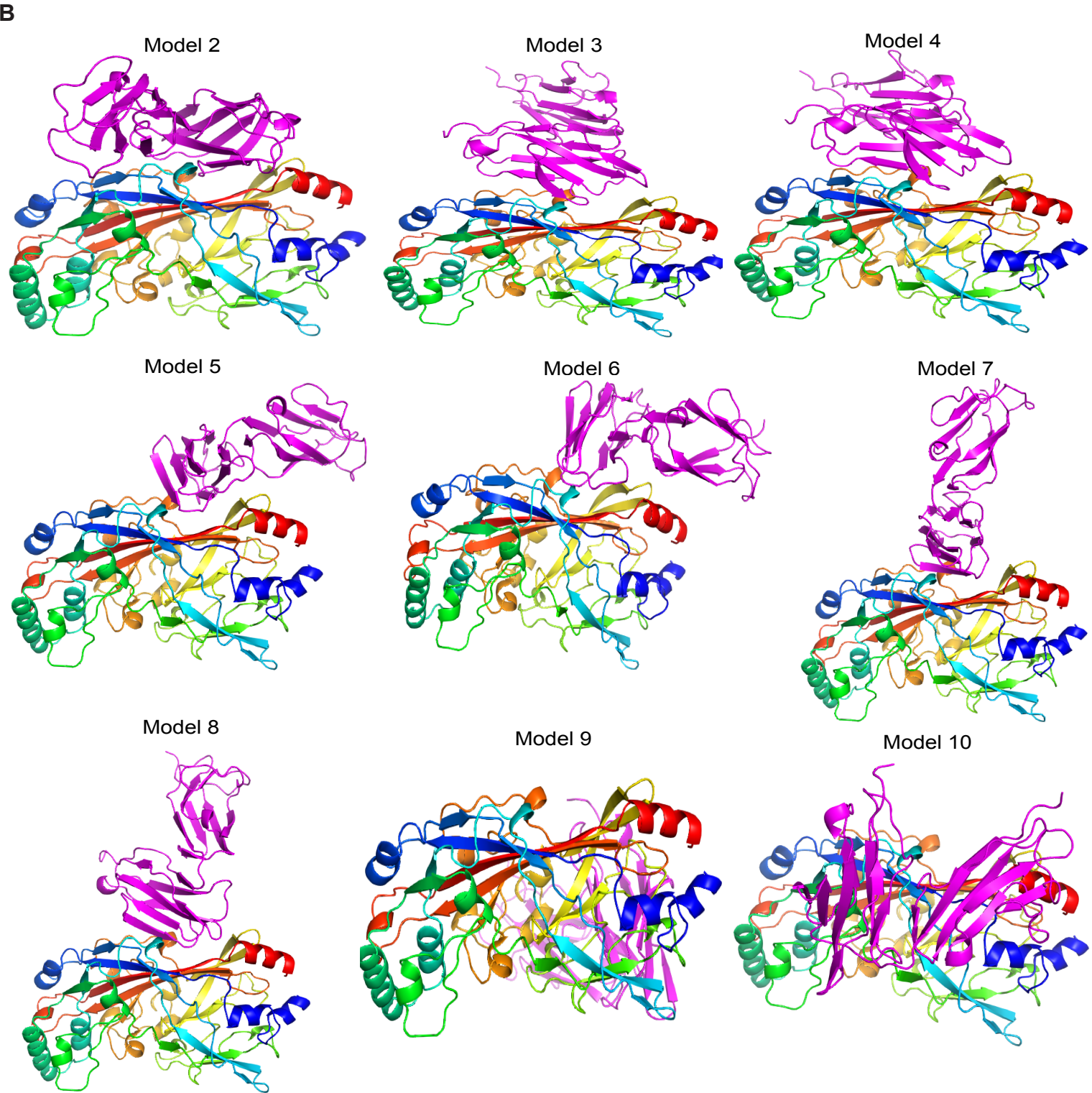

Supplement: Supplementary file 3 — Supplementary Material 3: Fig. S3. Immunoprecipitation by anti-FcγRIIb antibodies and models for the docking of CD36 with FcγRIIb. (A) Immunoprecipitation using anti-FcγRIIb antibodies. CPG-stimulated B cell protein served as the loading control, referred to as Input (Inp). The B cell protein was immunoprecipitated using isotype control IgG antibodies (IgG), and anti-FcγRIIb antibodies (FcγRIIb). (B) Models 2–10 depict the docking of CD36 with FcγRIIb on the front side. Purple and multicolor represent FcγRIIb and CD36 structure models, respectively. [file 11658_2024_593_MOESM3_ESM.pdf]
